# Visualization of anomalous origin and course of coronary arteries in 748 consecutive symptomatic patients by 64-slice computed tomography angiography

**DOI:** 10.1186/1471-2261-9-54

**Published:** 2009-12-11

**Authors:** Franz von Ziegler, Marco Pilla, Lori McMullan, Prasad Panse, Alexander W Leber, Norbert Wilke, Alexander Becker

**Affiliations:** 1 Ludwig-Maximilians-University, Department of Cardiology, Grosshadern Campus, Munich, Germany; 2 University of Florida, College of Medicine, Division of Cardiology, Jacksonville, Florida, USA; 3 University of Florida, College of Medicine, Department of Radiology, Jacksonville, Florida, USA

## Abstract

**Background:**

Coronary artery anomalies (CAAs) are currently undergoing profound changes in understanding potentially pathophysiological mechanisms of disease. Aim of this study was to investigate the prevalence of anomalous origin and course of coronary arteries in consecutive symptomatic patients, who underwent cardiac 64-slice multidetector-row computed tomography angiography (MDCTA).

**Methods:**

Imaging datasets of 748 consecutive symptomatic patients referred for cardiac MDCTA were analyzed and CAAs of origin and further vessel course were grouped according to a recently suggested classification scheme by Angelini et al.

**Results:**

An overall of 17/748 patients (2.3%) showed CAA of origin and further vessel course. According to aforementioned classification scheme no Subgroup 1- (absent left main trunk) and Subgroup 2- (anomalous location of coronary ostium within aortic root or near proper aortic sinus of Valsalva) CAA were found. Subgroup 3 (anomalous location of coronary ostium outside normal "coronary" aortic sinuses) consisted of one patient with high anterior origin of both coronary arteries. The remaining 16 patients showed a coronary ostium at improper sinus (Subgroup 4). Latter group was subdivided into a right coronary artery arising from left anterior sinus with separate ostium (subgroup 4a; n = 7) and common ostium with left main coronary artery (subgroup 4b; n = 1). Subgroup 4c consisted of one patient with a single coronary artery arising from the right anterior sinus (RAS) without left circumflex coronary artery (LCX). In subgroup 4d, LCX arose from RAS (n = 7).

**Conclusions:**

Prevalence of CAA of origin and further vessel course in a symptomatic consecutive patient population was similar to large angiographic series, although these patients do not reflect general population. However, our study supports the use of 64-slice MDCTA for the identification and definition of CAA.

## Background

Coronary artery anomalies (CAAs) are still topic of intense discussions. This diverse group of congenital disorders is likely to show a broad variability of clinical manifestations as well as pathophysiological mechanisms of disease [1-3]. Diagnosis of CAA is usually established during invasive coronary angiography (ICA). However, due to the two-dimensional projectional nature of ICA, the visualization of a complex three-dimensional vessel course as well as clarification of the exact relationship to surrounding anatomical structures may be difficult and misinterpretation is reported in up to 50% of the cases [4,5]. Recent technical developments in contrast-enhanced cardiac multidetector row computed tomography angiography (MDCTA) introducing faster scanners currently provide non-invasive three-dimensional imaging of coronary arteries. Besides a high diagnostic accuracy for the detection of significant coronary heart disease (CHD), current guidelines suggest usefulness of this diagnostic imaging modality for the evaluation of aberrant coronary vessel courses [6]. Multiple studies throughout the years have shown that MDCTA even with older scanner technology is a reliable non-invasive technique to identify CAAs and define their further course [7-10]. Although CAAs lack clinical significance in the majority of these patients, certain anomalous patterns, like anomalous origin of a coronary vessel from the opposite sinus have been associated with sudden cardiac death and ischemic complications [1,11]. For the evaluation of prevalence and clinical characteristics as well as for comparison of different imaging modalities, an exact definition of CAAs is mandatory. Classification criteria for CAAs have been extensively discussed in literature, but to date no general accepted classification scheme exists. In a recent publication a comprehensive and systematic approach on anatomical patterns has been proposed by Angelini [12]. The lack of such a strict terminology in current literature may partly explain the differences in reported prevalence ranging from 0.3% in necropsy studies up to 5.64% in a cineangiogram evaluation of 1,950 patients [11,13-18]. Aim of this study was to investigate the prevalence of CAA in consecutive symptomatic patients, who underwent cardiac 64-slice multidetector-row computed tomography angiography (MDCTA) by applying this suggested classification scheme for comparability reasons. This study focuses only on anomalies of origin and further vessel course. Myocardial bridges which are surely present in more than 1% of the general population suggest that this may be a normal variant and were therefore not included [12,19].

## Methods

### Patients

Between November 2005 and February 2007, a total number of 748 (389 male, 359 female, mean age: 47.0 ± 12.3 years, age range: 8-85 years) consecutive symptomatic patients were referred to the University of Florida, Department of Radiology, Shands, Jacksonville for cardiac MDCTA due to suspicion or assumed progression of CHD. Within these patients all datasets were reviewed in search of coronary anomalies of origin and further vessel course. General exclusion criteria for MDCTA according to the hospitals clinical practice were 1) unstable clinical conditions and inability to perform a short 10- to 15-second breathhold; 2) severe cardiac arrhythmias prior to the scan (e.g. bigeminy, trigeminy, and atrial fibrillation) known to cause severe image artifacts in MDCTA; 3) contraindications for a betablocker treatment, such as severe atrio-ventricular conduction blockage; 4) elevated serum markers suggesting myocardial infarction (Troponin); 5) renal function impairment (serum creatinine >1.5 mg/dl); and 6) known allergy to radiographic contrast media without a previous prophylactic medical treatment. Data analysis was approved by the institutional review board and patients gave written informed consent.

### MDCTA technique

Standardized patient preparation procedure included the administration of i.v.-betablocker (Metoprolol Tartrate, Bedford Labs, Bedford, OH) up to 10 mg prior to the scan in order to stabilize and/or lower their heart rates below 65 beats per minute if needed. Additionally, patients sublingually received Nitroglycerin (NitroQuick, Ethex Corp., St. Louis, MO) up to 800 μg immediately before contrast enhanced scan procedure to widen coronary arteries.

For MDCTA a 64-slice scanner (Sensation 64 Cardiac, Siemens Healthcare, Malvern, PA) was used. A native, prospectively ECG-triggered scan for coronary artery calcium scoring (CS) was performed first, followed by a contrast-enhanced, retrospectively ECG-gated coronary MDCTA scan. For CS tube voltage was 120 kV at a current of 200 mAs. The MDCTA scan protocol included a tube voltage of 120 kV at a current of 850 mAs - 950 mAs. Pitch was 0.2. Gantry rotation was 330 ms with the use of a halfscan algorithm for image reconstruction. Bolus tracking in the ascending aorta at a threshold of 100 HU was performed for timing. An overall of 100 ml of contrast agent (Iopromide, Ultravist 370 mgI/ml, Berlex, Montville, NJ) was used intravenously at a flow-rate of 5 ml/s. Out of these initially obtained raw-data sets, standardized image reconstruction according to the hospitals practice were performed at 25%, 45%, and 65% of the RR-Interval, respectively and therefore, no ECG-triggered tube modulation was used for data acquisition. If necessary, additional reconstructions throughout the whole cardiac cycle were made. For image reconstruction a slice thickness of 0.75 mm with an increment of 0.5 was chosen and a medium smooth body kernel (B25f) was applied. Additionally, average radiation exposure (MDCTA and CS) was estimated using the individual dose length product (DLP) given in the scan protocol multiplied by 0.017 mSv mGy-1 cm-1 (i.e. region-specific normalized effective dose coefficient for chest examinations in MDCT) as suggested by the European Guidelines on Quality Criteria for Computed Tomography [20].

### Image analysis

All acquired MDCTA images were transferred to a dedicated CT 3D-postprocessing workstation (Leonardo, Siemens Healthcare, Malvern, PA). Maximum Intensity Projections (MIPs), curved Multiplanar Reformats (cMPRs), and Volume Rendering Technique (VRT) were performed by experienced radiologists to evaluate coronary arteries. In agreement with Angelini, nature and name of a specific coronary artery was assigned not according to the site of origin or proximal course, but according to the dependent myocardial territory [21]. Thereby, as the right coronary artery (RCA), the vessel providing blood flow to the right ventricular wall and as the left anterior descending coronary artery (LAD), the vessel supplying the anterior interventricular septum was defined. The left circumflex artery (LCX) feeds the free wall of the left ventricle on the obtuse margin of the heart. Coronary anomalies were classified depending on anomalies of origin and further vessel course [12]. Thereby the following types were subdivided if existing: a) Absence of left main trunk, i.e. a split origination of the left main coronary artery and the left circumflex ramus (Subgroup 1); b) anomalous location of the coronary ostium within the aortic root or near proper sinus of Valsalva (Subgroup 2); c) anomalous location outside "normal" coronary ostium (Subgroup 3); and d) anomalous location of the coronary ostium at improper sinus of Valsalva, which may involve joint origination or "single" coronary pattern (Subgroup 4).

### Statistical analysis

All statistical analyses were performed using the MedCalc software package (MedCalc Software; Version 7.0.0.4; Mariakerke; Belgium) on a desktop computer. To compare ages of male and female an unpaired Wilcoxon-Test (Mann-Whitney) was used. All numbers are given in mean ± standard deviation. Calculations were considered to be significant at a p-value of < 0.05.

## Results

Table 1 gives a detailed overview of patient characteristics. An overall of 748 datasets were analyzed and 17 patients (2.27%; 12 male, 5 female) with CAAs were identified. Mean age was 43.1 ± 19.1 years (range: 15-73 years). In 7/748 patients (0.9%; 4 male, 3 female) CAA was already known and detected either by ICA (n = 4; 1 male, 3 female) or echocardiography (n = 3; all male). All CAA-patients received i.v.-betablockers and sublingually nitroglycerin. Mean heart rate during scan procedure was 59.44 ± 8.06 bpm (range: 45-73 bpm) and mean scan duration 18 ± 1 s. Estimated mean effective dose was calculated as 22.35 ± 4.62 mSv (range: 11.45-28.81 mSv). All scans were performed without complications and image quality was diagnostic in all scans.

**Table 1:** Patient characteristics

all patients		n = 748;
- age [years]		47.0 ± 12.3 (range: 8-85)
gender	male:	n = 389; 52.0%
	female:	n = 359; 48.0%

age [years]	male:	45.8 ± 13.0 (range: 8-81);
	female:	48.3 ± 11.3 (range:19-85); sig. (p = 0.0018)

indication for MDCT:	- chest pain:	n = 726; 97.1%; (n = 6 with known CAA)
	- abnormal stress test:	n = 21; 2.8%
	- syncope:	n = 1; 0.1%; (with known CAA)

**CAA-patients**		n = 17;
- age [years]		43.1 ± 19.1 (range: 15-73); n.s. (p = 0.3232)

gender	male:	n = 12; 70.6%
	female:	n = 5; 29.4%

age [years]	male:	40.4 ± 18.9 (range: 15-64);
	female:	49.6 ± 20.1 (range: 19-73); n.s. (p > 0.10)

heart rate during scan [bpm]		59.4 ± 8.1 (range: 45-73)

estimated radiation exposure [mSv]		22.4 ± 4.6 (range: 11.5-28.8)

Betablocker		n = 17;
Nitroglycerin		n = 17;

CAA: coronary artery anomaly of origin in further vessel course; n.s.: not significantly; sig.: significantly

Indications for MDCTA were evaluation of chest-pain (n = 726; 97.1%) or myocardial ischemia related symptoms, such as syncope (n = 1; 0.13%) or abnormal stress-test (n = 21; 2.8%) in the whole study population. Female (mean age: 48.3 ± 11.3 years; range: 19-85 years) patients were significantly older than male (mean age: 45.8 ± 13.0 years; range: 8-81 years) patients (p = 0.0018). Comparing the age of all patients with indications for MDCTA to the age of patients with CAA no significant difference was found (p = 0.3232). Within the CAA group female individuals (mean age: 49.6 ± 20.1 years; range: 19-73 years) were not significantly older than male patients (mean age: 40.4 ± 18.9 years; range: 15-65 years; p > 0.10).

According to the proposed classification scheme [12] with respect to anomalies of origin and further vessel course, an overall of two main types of CAAs were described and grouped under aforementioned classification scheme (see Table 2).

**Table 2:** Results for detected coronary artery anomalies

**Subgroup 1**:	Absent left main trunk (split origination of LCA); *not found*
**Subgroup 2**:	Anomalous location of coronary ostium within aortic root or near proper aortic sinus of Valsalva (for each artery); *not found*

**Subgroup 3**:	Anomalous location of coronary ostium outside normal "coronary" aortic sinuses
	• High origin of coronary arteries: left main coronary artery and right coronary artery; normal termination
	▪ n = 1 (0.1%)
	▪ gender: female
	▪ age: 19 years
	▪ symptoms: syncope
	▪ known coronary artery anomaly
	▪ initial diagnosis: ICA

**Subgroup 4**:	Anomalous location of coronary ostium at improper sinus (which may involve joint origination or "single" coronary pattern)
	• Right coronary artery from left anterior Sinus of Valsalva with further vessel course between aorta and pulmonary trunk (n = 8; 1.1%)
	○ separate ostium with left main coronary artery, normal termination (Subgroup 4a)
	▪ n = 7 (0.9%)
	▪ male:female = 4:3
	▪ age: 43.7 ± 18.2 years (range: 17-73 years)
	▪ symptoms: chest-pain
	▪ known coronary artery anomaly (n = 3)
	▪ initial diagnosis: ICA (n = 1); echocardiography (n = 2)
	○ common ostium with left main coronary artery, single coronary artery, normal termination (Subgroup 4b)
	▪ n = 1 (0.1%)
	▪ male
	▪ age: 26 years
	▪ symptoms: chest-pain
	▪ unknown coronary artery anomaly
	• Left coronary artery from right anterior Sinus of Valsalva with further vessel course between aorta and pulmonary trunk
	○ Common ostium with RCA and no circumflex ramus, single coronary artery, intramyocardial course of proximal left anterior descending coronary artery (Subgroup 4c)
	▪ n = 1 (0.1%)
	▪ gender: male
	▪ age: 58 years
	▪ symptoms: chest-pain
	▪ known coronary artery anomaly
	▪ initial diagnosis: ICA
	• Circumflex ramus from right anterior Sinus of Valsalva with further vessel course in posterior atrio-ventricular groove (Subgroup 4d)
	▪ n = 7 (0.94%)
	▪ male:female = 6:1
	▪ age: 46.3 ± 21.1 years (range: 15-65 years)
	▪ symptoms: chest-pain
	▪ known coronary artery anomaly (n = 1)
	▪ initial diagnosis: echocardiograpy (n = 1)

No patient with a Subgroup 1 (Absence of left main trunk) or Subgroup 2-CAA was found.

**Figure 1:**
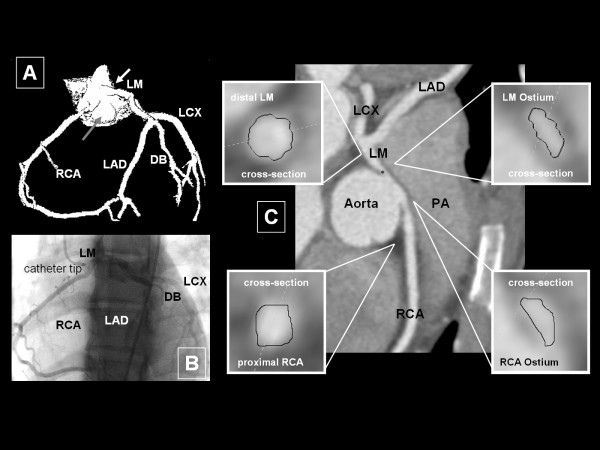
**High origin of the left and anterior origin of the right coronary artery (Subgroup 3)**. In this complex case a high origin of LM above the commissure between right and left coronary sinuses within the aortic root was reported. Furthermore RCA originates in a somewhat anterior position. Image A (Volume Rendering Technique) depicts the acute angle of LM (white arrow) above the aortic cusp (grey arrow), which is suspected as a possible mechanism of ischemia. In image B the close proximity of both coronary ostia in ICA is shown. Curved Multiplan Reformatting (Image C) displays further proximal course of LM and RCA between aorta and pulmonary artery. Note the ovoid cross sections of both intramural courses (cross-sectional images of RCA and LM), which is suspicious of lateral compression that may result in further compression during each systole especially under exercise conditions. DB: diagonal branch; LCX: left circumflex ramus; LA: left atrium; LAD: left anterior descending coronary artery; LM: left main coronary artery; PA: pulmonary artery; RCA: right coronary artery.

In 1/748 patients (0.1%) a complex anomalous coronary anatomy was investigated. Thereby inter-arterial (between aorta and pulmonary artery) and intramural courses for both left main coronary artery (LM) and RCA with a somewhat anterior position of the RCA-ostium within the right sinus of Valsalva was noted (see Figure 1; Subgroup 3). Further vessel course was unremarkable. This patient has had history of syncope related to exercise without chest-pain. Elevated cardiac enzymes (Troponin) raised suspicion of myocardial ischemia. A previous ICA confirmed an existing CAA. MDCTA was ordered for pre-surgical planning.

The remaining 16/748 patients (2.1%) were noted to present anomalous locations of the coronary orifices at improper sinuses (Subgroup 4). For a detailed overview, see Table 2.

**Figure 2:**
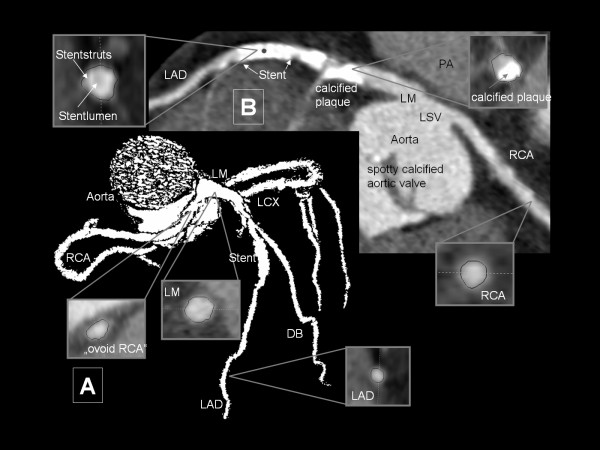
**RCA arising from left sinus of Valsalva with a separate ostium (Subgroup 4a)**. Image A (Volume Rendering Technique) depicts the whole coronary artery tree. RCA and LM are originating from the left sinus of Valsalva (LSV) with separate ostia (as shown in Image B, curved Multiplane Reformatting). Again note the ovoid cross-sectional image of the proximal intramural RCA course (left cross-sectional picture of Image A). Additionally, this patient obviously underwent stent implantation procedure (stent in mid LAD with good contrast enhancement within the stent lumen) due to CHD. Furthermore note the bright calcified plaque proximal to the previously implanted stent. This severe calcification causes so-called "blurring" impairing the luminal view. A high grade stenosis therefore cannot be ruled out. Interestingly, proximal LAD and RCA do not show any additional atherosclerotic plaque formation as depicted in the remaining cross-sectional images. Furthermore small calcified deposits (spotty calcification) are found at the aortic valve leaflets. LCX: left circumflex ramus; DB: diagonal branch; LAD: left anterior descending coronary artery; LM: left main coronary artery; LSV: left sinus of Valsalva; PA: pulmonary artery; RCA: right coronary artery.

**Figure 3:**
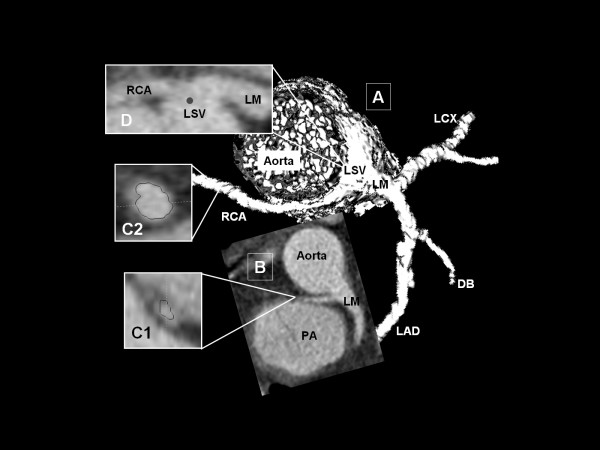
**Single coronary artery originating from left sinus of Valsalva (Subgroup 4b)**. This example illustrates a single coronary artery arising from the left coronary sinus of Valsalva with further intramural proximal course RCA (Image A; Volume Rendering Technique). The axial slice nicely depicts the close proximity of RCA and pulmonary artery (Image B). Again proximal RCA appears elliptical suspicious of lateral compression (Image C1, cross-sectional image) widening up after its intramural course (Image C2, cross-sectional image). Curved Multiplan Reformat shows the common ostium of left main coronary artery and RCA (Image D). DB: diagonal branch, LCX: left circumflex coronary artery, LM: left main coronary artery; LSV: left sinus of Valsalva; PA: pulmonary artery; RCA: right coronary artery.

In 8 patients (1.1%) the RCA arose from the opposite sinus of Valsalva with a separate ostium for RCA and LM in 7 cases (Subgroup 4a; 0.9%, Figure 2) and a common ostium of RCA and LM (single coronary artery) in one case (Subgroup 4b; 0.1%; see Figure 3). All of them (5 male; 3 female) experienced symptoms of chest pain and showed proximal intramural courses of the RCA, but with unremarkable termination. Mean age was 41.5 ± 18.0 years (range: 17-73 years). An overall of 4 patients already were diagnosed as having CAAs, either with previous out-clinic ICA (n = 2) or echocardiography (n = 2).

**Figure 4:**
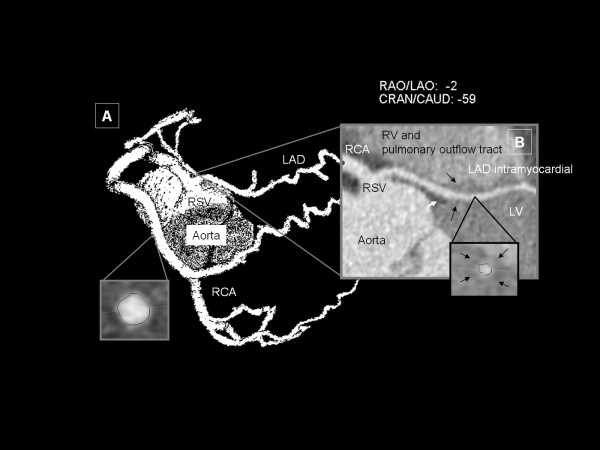
**Single coronary artery originating from right sinus of Valsalva (Subgroup 4c)**. This case shows a single coronary artery arising from right sinus of Valsalva (common ostium of RCA and LAD without circumflex ramus) in a Maximum Intensity Projection (Image A). Myocardial territory usually supplied by LCX is fed by RCA (right dominant type) and LAD is noted to run intra-myocardial within the left ventricular septum (Image B; Curved Multiplane Reformatting). Note the surrounding muscular tissue (also depicted in the cross-sectional image of LAD) marked with black arrows which appears lighter grey compared to epicardial adipose tissue (white arrow). LAD: left anterior descending coronary artery; LV: left ventricle; RA: right atrium; RCA: right coronary artery; RSV: right sinus of Valsalva; RV: right ventricle.

One patient (Subgroup 4c; 0.1%; 58 year old male) had an anomalous origination of LM from the opposite sinus together with RCA (common ostium; single coronary artery) as depicted in Figure 4. Additionally the proximal part of the left anterior descending coronary artery (LAD) was noted to course intramyocardially ("myocardial bridge"). Termination was found to be normal. Small coronary calcifications were present in proximal parts of the RCA. This patient also complained of chest pain and a previous out-clinic ICA verified the existence of a CAA and furthermore non-stenotic CHD. The indication for cardiac MDCTA in this specific case was ruling out of progression of CHD and further clarification of anatomical relationships.

**Figure 5:**
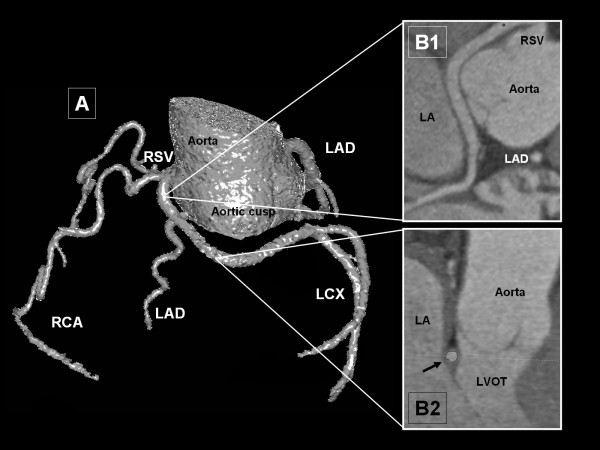
**Circumflex ramus originating from right sinus of Valsalva with further posterior vessel course (Subgroup 4d)**. This case shows an abnormal origin of LCX from the right sinus of Valsalva with a further posterior (retroaortic) course of LCX within the atrioventricular groove (Image A, Volume Rendering Technique, posterior view). Cross-sectional curved Multiplane Reformats nicely depict the anatomic relationships of the vessel, left atrium and Aorta (Images B). In Image B2 the retroaortic course within the atrioventricular groove of LCX is marked with a black arrow. LCX: left circumflex ramus; LA: left atrium; LAD: left anterior descending coronary artery; LVOT: left ventricular outflow tract; RCA: right coronary artery; RSV: right sinus of Valsalva.

The remaining 7 patients (6 male; 1 female) showed an abnormal origin of LCX from the right sinus of Valsalva with a further posterior course within the atrioventricular groove (Subgroup 4d; 0.9%; see Figure 5). In latter cases no additional anomalous courses of LAD were depicted. Termination of LCX was normal in all patients. Coronary calcifications were present in an overall of 4 patients.

## Discussion

According to the current literature, CAAs occur in roughly 1% of the general population. This prevalence is derived from ICA studies performed for suspected CHD [14-16,18]. Necropsy studies report even lower numbers: Alexander and Griffith [13] observed only 54 CAAs in 18,950 cases (0.3%). These studies are limited by entry bias and lack of clear diagnostic criteria, which both are prerequisites for defining the true prevalence in a general population. The first study adopting strict criteria for assessing CAAs was done by Angelini and co-workers. They prospectively analyzed 1,950 consecutive cineangiograms [11]. Thereby the authors reported a prevalence for CAAs of 5.6%, which is higher than the usually cited prevalence derived from angiographic reports, but comparable to one of the first reports using 64-slice CT in a somehow similar approach. In this study the authors report a prevalence of coronary anomalies of origin and further course of 7.9% in mainly symptomatic patients [22]. De Jonge and co-workers also describe a prevalence of 7% of CAAs including coronary fistulas [10] in their patient population. These discrepancies in reported prevalence might be caused by referral bias. Some of these patients with CAAs might have been or were referred because of known presence of CAA and not because of unrelated factors as in the general population. In our study an overall of 17 patients (2.3%) with CAAs were identified. 41.2% of these patients (n = 7) were already diagnosed as having CAAs by other imaging modalities, such as ICA or echocardiography. Excluding these patients, leads to a prevalence of an anomalous coronary vessel origination of 1.4% in symptomatic patients. This result is quite similar to that observed in a large angiographic series [18], as well as in two large previously published MDCTA studies dealing either with 4- and 16-slice CT scanner technology and including 1758 patients [23] or with 64-slice CT in 1495 patients [24]. However, even such large studies do not reflect general population as only symptomatic patients with indications for either ICA or MDCTA were considered.

In our study, where roughly the same number of men and women were initially examined, CAAs appear to be more common in men (n = 12; 71%) than in women (n = 5; 29%). This was also shown in previous reports [14,25,26], although such a finding may reflect the selective nature of referral for cardiac MDCTA. The most common coronary anomaly in our patient population was an anomalous RCA arising from the opposite sinus of Valsalva (n = 8; 1.1%), followed by an anomalous origin of the circumflex ramus (n = 7; 0.9%). These findings are similar to previously published angiographic studies [11,16,27], although Wilkins et al [25], as well as Yamanaka et al [18], in the largest angiographic trial including 126,595 patients, report different prevalence in their study population. Interestingly, we did not find any split left main coronary artery (Subgroup 1) or a CAA within the aortic root near proper sinus of Valsalva (Subgroup 2), which might be explainable by referral bias and presumably lack of ischemia related symptoms in these specific subgroups as only symptomatic patients were examined in our population. Nevertheless these inconsistent findings concerning the prevalence of CAA and, moreover, different subgroups suggest that the described numbers are only true for our study population. Therefore, a general conclusion for asymptomatic individuals cannot be drawn.

The clinical impact of CAAs still remains controversial. Coronary anomalies cause up to 17% of deaths in athletes, and furthermore are associated with 12% of sport-related deaths in 14- to 40-year-old individuals [17,28,29]. Furthermore, anomalous origination of a coronary artery from the opposite sinus is related to sudden death as reported in frequently quoted autopsy reports [30]. In other anomalies, ischemia occurs only under inconsistent or extreme clinical conditions. In our study population, all patients had either chest-pain or previous history of syncope related to exercise independently of the type of detected CAA. But out of these findings in a small symptomatic patient cohort, no conclusion regarding the malignancy of CAAs in relation to morphological characteristics can be drawn.

### Limitations

Although, for screening purposes in order to define the true prevalence, non-invasive imaging modalities should be considered and ECG-gated contrast-enhanced MDCTA has been shown to accurately identify and therefore is considered to be a reliable method for evaluation of CAAs [6,31], it also adhere inevitable risks: utilization of ionizing radiation and the need of iodinate contrast agent application. In our study estimated mean effective dose was calculated as 22.35 ± 4.62 mSv, which is three to four times higher than in diagnostic ICA [32]. Therefore, increasing interest focus on radiation dose reduction tools, like ECG-controlled tube modulation or prospectively ECG-triggered image acquisition. Initial experiences raise hope that the use of advanced imaging protocols in Dual-Source CT or 320-slice CT may lead to reduced radiation exposure [33-35]. Taking into consideration, that screening for CAAs mainly involves a younger population; MDCTA to date might not be a useful tool for this purpose [36]. In this respect, cardiac magnetic resonance tomography (CMR) which offers excellent diagnostic accuracies compared to ICA, should be considered as an alternative test [6].

Additionally, the results of our study were derived by a symptomatic patient cohort with indications for MDCTA in a single center study, so that our results are also biased by referral and do not reflect a general population. Furthermore, this study consisted of 748 patients in which no Subgroup 1- and Subgroup 2-CAA were found suggesting that the study population, although only CAAs of origin and further vessel course were observed is too small.

## Conclusions

This study applies a strict classification scheme for detecting CAAs of origin and further vessel course in a symptomatic consecutive patient population utilizing cardiac 64-slice MDCTA and supports the use of CT technology for the identification and definition of CAA. Prevalence of these CAAs was similar to large angiographic studies. However, our study population does not represent a general population. But for screening purpose in asymptomatic patients other imaging modalities, such as CMR should be considered.

## Competing interests

The authors declare that they have no competing interests.

## Authors' contributions

FvZ is responsible for coordination of the study, statistical evaluation and manuscript. MP and LM are responsible for the evaluation of MDCTA data sets. PP performed MDCTA scanning and helped to draft the manuscript. AL contributed in conception and design of the study. NW supervised MDCTA data evaluation and interpretation. AB is responsible for manuscript review.

All authors have read and approved the final manuscript.

## Pre-publication history

The pre-publication history for this paper can be accessed here:

http://www.biomedcentral.com/1471-2261/9/54/prepub

## References

[B1] Basso C Maron BJ Corrado D Thiene G Clinical profile of congenital coronary artery anomalies with origin from the wrong aortic sinus leading to sudden death in young competitive athletes J Am Coll Cardiol 2000 35 1493 1501 10.1016/S0735-1097(00)00566-0 10807452

[B2] Liberthson RR Sudden death from cardiac causes in children and young adults N Engl J Med 1996 334 1039 1044 10.1056/NEJM199604183341607 8598843

[B3] Maron BJ Shirani J Poliac LC Mathenge R Roberts WC Mueller FO Sudden death in young competitive athletes. Clinical, demographic, and pathological profiles JAMA 1996 276 199 204 10.1001/jama.276.3.199 8667563

[B4] Ishikawa T Brandt PW Anomalous origin of the left main coronary artery from the right anterior aortic sinus: angiographic definition of anomalous course Am J Cardiol 1985 55 770 776 10.1016/0002-9149(85)90154-7 3976523

[B5] Shi H Aschoff AJ Brambs HJ Hoffmann MH Multislice CT imaging of anomalous coronary arteries Eur Radiol 2004 14 2172 2181 10.1007/s00330-004-2490-2 15490179

[B6] Bluemke DA Achenbach S Budoff M Gerber TC Gersh B Hillis LD Hundley WG Manning WJ Printz BF Stuber M Woodard PK Noninvasive coronary artery imaging: magnetic resonance angiography and multidetector computed tomography angiography: a scientific statement from the american heart association committee on cardiovascular imaging and intervention of the council on cardiovascular radiology and intervention, and the councils on clinical cardiology and cardiovascular disease in the young Circulation 2008 118 586 606 10.1161/CIRCULATIONAHA.108.189695 18586979

[B7] Datta J White CS Gilkeson RC Meyer CA Kansal S Jani ML Arildsen RC Read K Anomalous coronary arteries in adults: depiction at multi-detector row CT angiography Radiology 2005 235 812 818 10.1148/radiol.2353040314 15833984

[B8] van Ooijen PM Dorgelo J Zijlstra F Oudkerk M Detection, visualization and evaluation of anomalous coronary anatomy on 16-slice multidetector-row CT Eur Radiol 2004 14 2163 2171 10.1007/s00330-004-2493-z 15452665

[B9] Schmid M Achenbach S Ludwig J Baum U Anders K Pohle K Daniel WG Ropers D Visualization of coronary artery anomalies by contrast-enhanced multi-detector row spiral computed tomography Int J Cardiol 2006 111 430 435 10.1016/j.ijcard.2005.08.027 16271776

[B10] de Jonge GJ van Ooijen PM Piers LH Dikkers R Tio RA Willems TP Heuvel AF van den Zijlstra F Oudkerk M Visualization of anomalous coronary arteries on dual-source computed tomography Eur Radiol 2008 18 2425 2432 10.1007/s00330-008-1110-y 18651148

[B11] Angelini P Velasco JA Flamm S Coronary anomalies: incidence, pathophysiology, and clinical relevance Circulation 2002 105 2449 2454 10.1161/01.CIR.0000016175.49835.57 12021235

[B12] Angelini P Coronary artery anomalies: an entity in search of an identity Circulation 2007 115 1296 1305 17353457 10.1161/CIRCULATIONAHA.106.618082

[B13] Alexander RW Griffith GC Anomalies of the coronary arteries and their clinical significance Circulation 1956 14 800 805 13374855 10.1161/01.cir.14.5.800

[B14] Chaitman BR Lespérance J Saltiel J Bourassa MG Clinical, angiographic, and hemodynamic findings in patients with anomalous origin of the coronary arteries Circulation 1976 53 122 131 1244233 10.1161/01.cir.53.1.122

[B15] Engel HJ Torres C Page HL Jr Major variations in anatomical origin of the coronary arteries: angiographic observations in 4,250 patients without associated congenital heart disease Cathet Cardiovasc Diagn 1975 1 157 169 10.1002/ccd.1810010205 1222415

[B16] Topaz O DeMarchena EJ Perin E Sommer LS Mallon SM Chahine RA Anomalous coronary arteries: angiographic findings in 80 patients Int J Cardiol 1992 34 129 138 10.1016/0167-5273(92)90148-V 1737663

[B17] von Kodolitsch Y Franzen O Lund GK Koschyk DH Ito WD Meinertz T Coronary artery anomalies Part II: recent insights from clinical investigations Z Kardiol 2005 94 1 13 10.1007/s00392-005-0153-1 15668824

[B18] Yamanaka O Hobbs RE Coronary artery anomalies in 126,595 patients undergoing coronary arteriography Cathet Cardiovasc Diagn 1990 21 28 40 10.1002/ccd.1810210110 2208265

[B19] Zeina AR Odeh M Blinder J Rosenschein U Barmeir E Myocardial bridge: evaluation on MDCT AJR Am J Roentgenol 2007 188 1069 1073 10.2214/AJR.06.0714 17377049

[B20] Bongartz G Golding SJ Jurik AG Leonardi M van Meerten EvP Geleijns J Jessen KA Panzer W Shrimpton PC Tosi G European Guidelines on Quality Criteria for Computed Tomography EUR 16262 EN 1999 http://www.drs.dk/guidelines/ct/quality/mainindex.htm Accessed Oct 16 2009

[B21] Angelini P Coronary artery anomalies--current clinical issues: definitions, classification, incidence, clinical relevance, and treatment guidelines Tex Heart Inst J 2002 29 271 278 12484611 PMC140289

[B22] Cademartiri F La Grutta L Malagò R Alberghina F Meijboom WB Pugliese F Maffei E Palumbo AA Aldrovandi A Fusaro M Brambilla V Coruzzi P Midiri M Mollet NR Krestin GP Prevalence of anatomical variants and coronary anomalies in 543 consecutive patients studied with 64-slice CT coronary angiography Eur Radiol 2008 18 781 791 10.1007/s00330-007-0821-9 18246357 PMC2270369

[B23] Schmitt R Froehner S Brunn J Wagner M Brunner H Cherevatyy O Gietzen F Christopoulos G Kerber S Fellner F Congenital anomalies of the coronary arteries: imaging with contrast-enhanced, multidetector computed tomography Eur Radiol 2005 15 1110 1121 10.1007/s00330-005-2707-z 15756551

[B24] Srinivasan KG Gaikwad A Kannan BR Ritesh K Ushanandini KP Congenital coronary artery anomalies: diagnosis with 64 slice multidetector row computed tomography coronary angiography: a single-centre study J Med Imaging Radiat Oncol 2008 52 148 154 10.1111/j.1440-1673.2008.01933.x 18373806

[B25] Wilkins CE Betancourt B Mathur VS Massumi A De Castro CM Garcia E Hall RJ Coronary artery anomalies: a review of more than 10,000 patients from the Clayton Cardiovascular Laboratories Tex Heart Inst J 1988 15 166 173 15227247 PMC324820

[B26] Kimbiris D Anomalous origin of the left main coronary artery from the right sinus of Valsalva Am J Cardiol 1985 55 765 769 10.1016/0002-9149(85)90153-5 3976522

[B27] Garg N Tewari S Kapoor A Gupta DK Sinha N Primary congenital anomalies of the coronary arteries: a coronary: arteriographic study Int J Cardiol 2000 74 39 46 10.1016/S0167-5273(00)00243-6 10854679

[B28] Burke AP Farb A Virmani R Goodin J Smialek JE Sports-related and non-sports-related sudden cardiac death in young adults Am Heart J 1991 121 568 575 10.1016/0002-8703(91)90727-Y 1825009

[B29] Maron BJ Gohman TE Aeppli D Prevalence of sudden cardiac death during competitive sports activities in Minnesota high school athletes J Am Coll Cardiol 1998 32 1881 1884 10.1016/S0735-1097(98)00491-4 9857867

[B30] Taylor AJ Rogan KM Virmani R Sudden cardiac death associated with isolated congenital coronary artery anomalies J Am Coll Cardiol 1992 20 640 647 1512344 10.1016/0735-1097(92)90019-j

[B31] Coles DR Smail MA Negus IS Wilde P Oberhoff M Karsch KR Baumbach A Comparison of radiation doses from multislice computed tomography coronary angiography and conventional diagnostic angiography J Am Coll Cardiol 2006 47 1840 1845 10.1016/j.jacc.2005.11.078 16682310

[B32] Hendel RC Patel MR Kramer CM Poon M Hendel RC Carr JC Gerstad NA Gillam LD Hodgson JM Kim RJ Kramer CM Lesser JR Martin ET Messer JV Redberg RF Rubin GD Rumsfeld JS Taylor AJ Weigold WG Woodard PK Brindis RG Hendel RC Douglas PS Peterson ED Wolk MJ Allen JM Patel MR American College of Cardiology Foundation Quality Strategic Directions Committee Appropriateness Criteria Working Group American College of Radiology Society of Cardiovascular Computed Tomography Society for Cardiovascular Magnetic Resonance American Society of Nuclear Cardiology North American Society for Cardiac Imaging Society for Cardiovascular Angiography and Interventions Society of Interventional Radiology ACCF/ACR/SCCT/SCMR/ASNC/NASCI/SCAI/SIR 2006 appropriateness criteria for cardiac computed tomography and cardiac magnetic resonance imaging: a report of the American College of Cardiology Foundation Quality Strategic Directions Committee Appropriateness Criteria Working Group, American College of Radiology, Society of Cardiovascular Computed Tomography, Society for Cardiovascular Magnetic Resonance, American Society of Nuclear Cardiology, North American Society for Cardiac Imaging, Society for Cardiovascular Angiography and Interventions, and Society of Interventional Radiology J Am Coll Cardiol 2006 48 1475 1497 10.1016/j.jacc.2006.07.003 17010819

[B33] Rybicki FJ Otero HJ Steigner ML Vorobiof G Nallamshetty L Mitsouras D Ersoy H Mather RT Judy PF Cai T Coyner K Schultz K Whitmore AG Di Carli MF Initial evaluation of coronary images from 320-detector row computed tomography Int J Cardiovasc Imaging 2008 24 535 546 10.1007/s10554-008-9308-2 18368512

[B34] Stolzmann P Scheffel H Schertler T Frauenfelder T Leschka S Husmann L Flohr TG Marincek B Kaufmann PA Alkadhi H Radiation dose estimates in dual-source computed tomography coronary angiography Eur Radiol 2008 18 592 599 10.1007/s00330-007-0786-8 17909816

[B35] Husmann L Valenta I Gaemperli O Adda O Treyer V Wyss CA Veit-Haibach P Tatsugami F von Schulthess GK Kaufmann PA Feasibility of low-dose coronary CT angiography: first experience with prospective ECG-gating Eur Heart J 2008 29 191 197 10.1093/eurheartj/ehm613 18089704

[B36] Zanzonico P Rothenberg LN Strauss HW Radiation exposure of computed tomography and direct intracoronary angiography: risk has its reward J Am Coll Cardiol 2006 47 1846 1849 10.1016/j.jacc.2005.10.075 16682311

